# Repair of a Large Vermilion Defect of the Lower Lip Following Micrographic Surgery

**DOI:** 10.7759/cureus.84128

**Published:** 2025-05-14

**Authors:** Garrett P Kraft, Harib H Ezaldein

**Affiliations:** 1 Dermatology, Miami Dermatology and Mohs Surgery, Miami, USA; 2 Dr. Phillip Frost Department of Dermatology and Cutaneous Surgery, University of Miami Miller School of Medicine, Miami, USA; 3 Mohs Micrographic Surgery, Bennett Surgery Center, Santa Monica, USA

**Keywords:** facial surgery, lip cancer, lower lip, mohs surgery, post mohs wound, squamous cell carcinoma (scc)

## Abstract

Reconstruction of surgical defects of the face following Mohs micrographic surgery (MMS) requires careful consideration of both functional and cosmetic outcomes. In this report, we present a complex case of biopsy-proven invasive Bowenoid squamous cell carcinoma with extensive lower lip involvement, resulting in a large musculomucosal defect after MMS. The defect was closed using a multilayered approach with local tissue advancement to reduce tension and eliminate the need for secondary donor sites. Long-term follow-up demonstrated excellent cosmetic and functional outcomes without evidence of recurrence.

## Introduction

Mohs micrographic surgery (MMS) is a highly effective technique for treating cutaneous malignancies. It offers superior cure rates while maximizing the preservation of healthy tissue. It is considered the gold standard for managing high-risk basal cell carcinoma and squamous cell carcinoma (SCC), particularly in anatomically sensitive areas such as the face [1].

The lips serve both functional and aesthetic roles, making malignancies in this area especially challenging. Involvement of the lips can impair speech production, oral competence, and facial expression [2]. The lower lip is the most common site of lip SCC, likely due to chronic UV exposure, with incidence highest in fair-skinned men over age 50. While often diagnosed at an early stage, lip SCC carries a variable risk of nodal metastasis (10-19%) and a five-year survival rate of approximately 90-92% with surgical treatment. It has been suggested that lip SCC behaves more aggressively than other cutaneous SCCs of the head and neck [3].

Reconstruction following MMS defects of the lip must balance both form and function. Defects involving the lower lip often require complex reconstruction techniques to prevent complications such as microstomia, oral incompetence, and aesthetic distortion [2]. Various approaches, including primary closure, local flaps, mucosal advancements, and grafts, have been described depending on defect size, depth, and location. However, large vermilion defects pose additional challenges due to limited available tissue, risk of tension-related dehiscence, and the need to maintain adequate mobility and sensation [4].

This report presents a case of extensive lower lip vermilion involvement by invasive Bowenoid SCC requiring MMS. The procedure was followed by a multilayered reconstructive approach designed to preserve lip function, minimize tension, and optimize cosmetic outcomes.

## Case presentation

**Figure 1:**
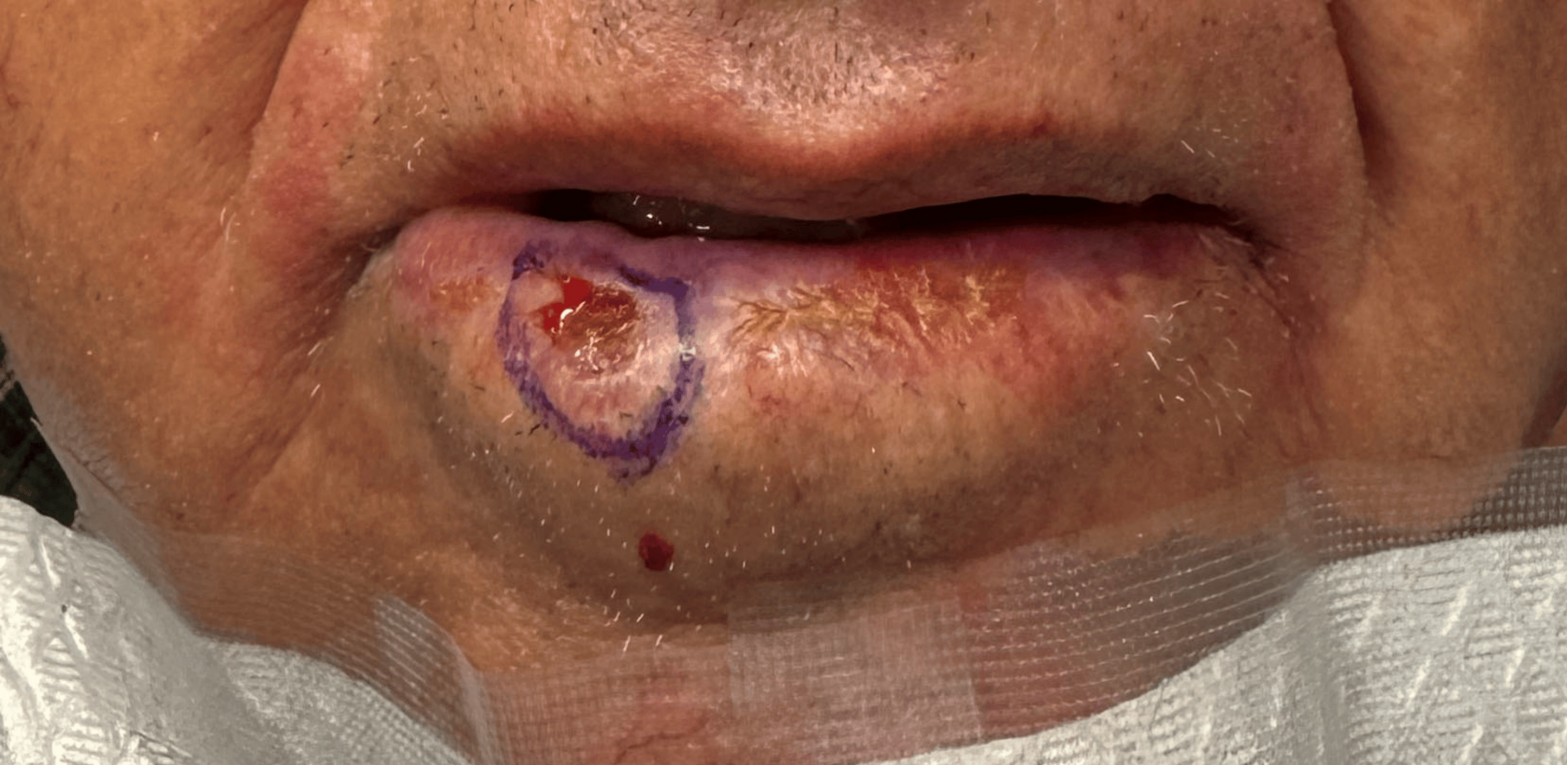
Clinical presentation of invasive Bowenoid SCC prior to Mohs surgery The lesion is outlined with a surgical marking pen to delineate visible tumor margins prior to excision. SCC: squamous cell carcinoma

An 80-year-old man presented with a biopsy-proven invasive Bowenoid SCC initially measuring 1.5 × 1.2 cm on the right lower lip vermilion (Figure 1). The lesion first appeared as a growing, scaly, pink papule and was treated multiple times with cryotherapy by an unlicensed provider prior to biopsy-proven diagnosis. The patient then presented to his primary dermatologist, who performed a shave biopsy, and he was immediately referred for MMS. There were no clinically palpable lymph nodes or in-transit metastatic lesions at presentation.

**Figure 2:**
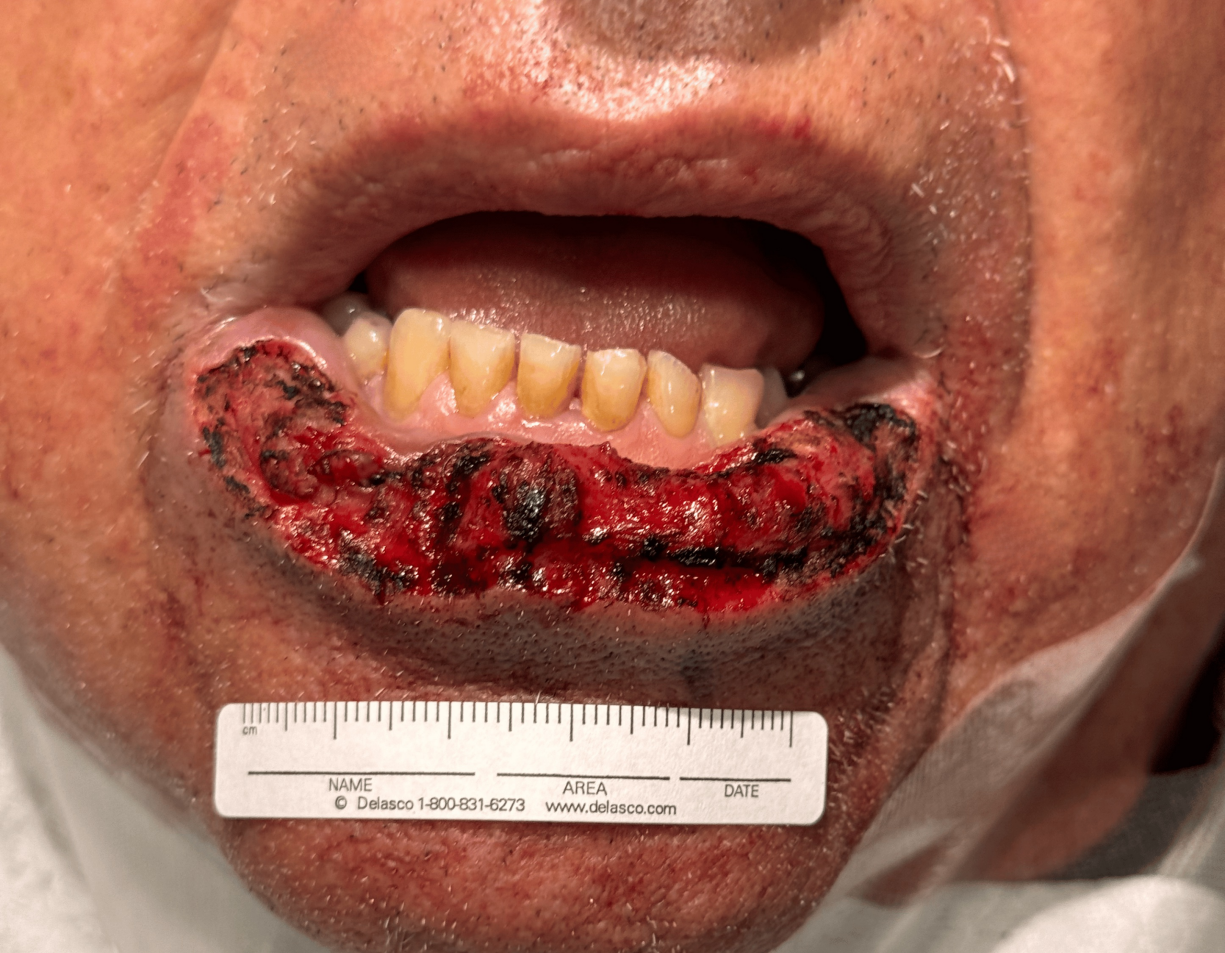
Surgical defect of the lower lip vermilion following tumor removal

MMS was completed in five stages, resulting in a final musculomucosal defect measuring 6.8 × 1.7 cm (Figure 2), encompassing approximately 90% of the lower lip surface area. The defect was confined to the dry vermilion and adjacent oral mucosa. Following tumor removal and prior to final reconstruction, the patient demonstrated intact labial and perioral sensation and function. Histological findings were significant for full-thickness epithelial atypia with invasion into the dermis and submucosa and increased mitotic figures. These findings were consistent with a diagnosis of invasive Bowenoid SCC.

**Figure 3:**
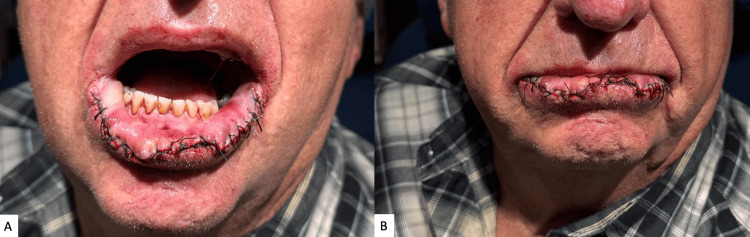
Immediate postoperative appearance following lip reconstruction with inverted subdermal-muscular plication and mucocutaneous advancement Mouth open demonstrating tension-free closure and preservation of oral commissure alignment (A). Mouth closed showing maintained lip contour and symmetry (B).

**Figure 4:**
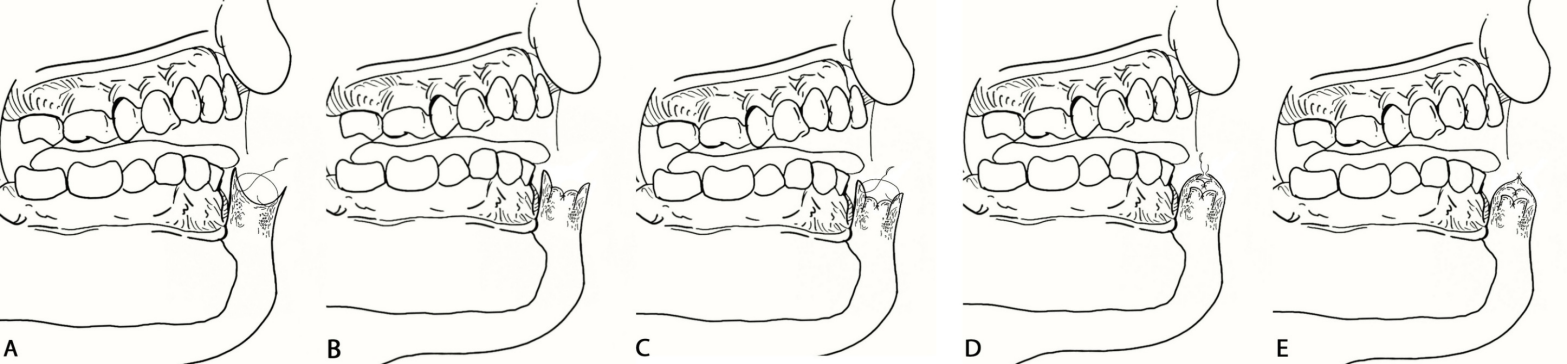
Illustrated sequence of plication technique for lower lip defect repair Placement of the first deep plication layer using inverted muscular-submucosal sutures (4-0 Vicryl) to approximate all tissue planes and redirect tension away from the epidermal closure (A). Completion of the plication layer demonstrating approximation of tissue layers and displacement of tension from superficial planes (B). Placement of the second plication layer across the submucosal and dermal interface using inverting 5-0 Vicryl sutures to advance the planes without the need for sulcal incision or backcut (C). Completion of the second plication layer, demonstrating close approximation of the mucocutaneous junction with minimal residual tension on the epidermal edges (D). Final closure using interrupted 5-0 silk sutures at the epidermal level, completing the triple-layer repair with restored lip contour and tension-free approximation (E). Image Credit: Author

We elected to repair the defect with an inverted subdermal-muscular-submucosal plication layer of sutures and a mucocutaneous advancement, without a sulcal incision or backcut. This triple-layer approach utilized deep inverting plication sutures (4-0 Vicryl) to reduce tension at the superficial dermal and submucosal layers when advanced together. The mucocutaneous advancement was then performed with minimal tension using simple interrupted vertical mattress sutures (5-0 Vicryl), and the epidermal edges were approximated with interrupted 5-0 silk sutures. This resulted in a final closure with minimized tension, decreased risk of wound dehiscence, and no need for secondary wound sites (Figure 3). The plication layer effectively displaced the tension required for advancing the mucosal and cutaneous edges to the deeper aspects of the lip (Figure 4).

**Figure 5:**
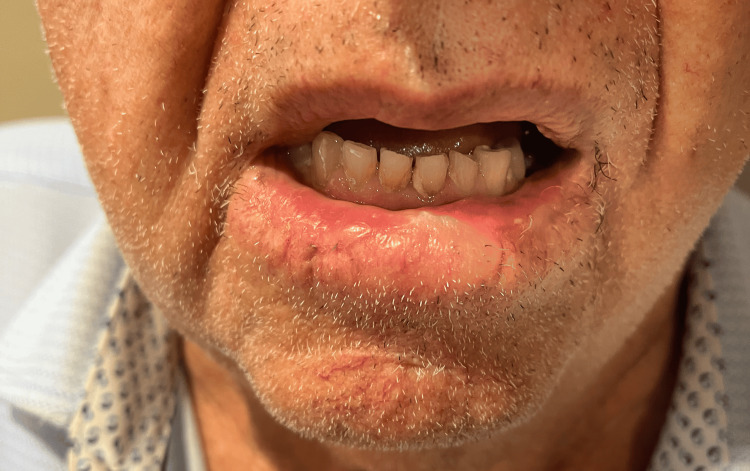
Postoperative result at six-month follow-up

The patient was given specific instructions regarding wound care, including a soft diet for one week during initial healing. He was also instructed to use chlorhexidine gluconate 0.12% oral rinses three times a day and petrolatum ointment applications around the clock. Sutures were removed two weeks postoperatively, and the patient was seen at six months with intact stomal function, a good color and texture match, and no evidence of contracture or microstomia (Figure 5).

## Discussion

Repairing surgical defects of the lip requires careful attention to maintaining lip structure and dimensions. The lips play a critical role in maintaining oral continence, facilitating facial expression, and supporting speech production. As a focal point of the face, lip reconstruction entails cosmetic concerns for patients. Achieving a simpler repair with minimal distortion or scarring is especially important given our patient’s advanced age and social circumstances, which may increase the risk of healing complications.

Various options were considered when choosing a method of repair. When the defect involves less than one-third of the horizontal width of the vermilion, primary wedge closure can be utilized to achieve optimal cosmetic and functional outcomes; however, this was not an option given the large size of the defect encompassing 90% of the lower lip [5]. Prior studies have confirmed that primary closure under excessive tension increases the risk of microstomia, vermilion distortion, and functional compromise in lip reconstruction, particularly for defects greater than one-third of the lip width [6].

Healing by secondary intent was not an ideal option for this patient due to the depth and extent of the resection, which poses concerns for wound care compliance and suboptimal functional outcomes (e.g., microstomia and oral incontinence) [7]. However, retrospective case series have described secondary intention healing for small defects as having low complication rates and favorable cosmetic and functional outcomes [8].

The literature describes full-thickness skin grafts in this location with acceptable outcomes; however, concerns for this type of repair include irregular contour, poor color and texture match, a secondary wound site prone to bacteremia, and difficulties with immobilizing and keeping the graft dry [9]. Studies evaluating full-thickness grafting in mobile areas such as the lips have demonstrated inferior aesthetic outcomes compared to local flap techniques [10].

Repair with a local flap is indicated for defects measuring greater than one-third of the lower lip, with the advantage of like-with-like tissue replacement resulting in similar texture and appearance [11]. A mucosal advancement for this type of defect can be considered; however, there is an increased risk of dehiscence, and the secondary sulcus defect would significantly increase wound care requirements. Cutaneous and mucosal pedicle advancements or transposition flaps may also be considered. However, downsides include larger incision lengths, dehiscence risks, and shortening of the lateral dimensions of the lip (transposition flaps). Prior comparative studies suggest that flap-based reconstructions provide superior aesthetic results and dynamic function compared to grafting alone in large lower lip defects. Grafting has been associated with insufficient oral competence, causing salivary leakage and impaired upper and lower lip synchronous movement, leading to speech disturbances [12].

Ultimately, using an inverted subdermal-muscular plication layer of sutures and a mucocutaneous advancement without a sulcal incision or backcut, the chosen repair method achieved tension-minimized closure without any secondary wound sites. While not extensively described in existing literature, this layered method may offer a useful strategy for large central lip defects when primary closure or traditional flap techniques are not ideal. This closure technique may be considered whenever local tissue mobility permits plication without tension. While there are no strict limitations regarding defect size or depth, it is beneficial for defects where traditional mucosal advancement would risk disruption of the gingival sulcus or involve a sulcal incision. This approach is similar to other plication methods used elsewhere in the head and neck, offering a tissue-sparing alternative that preserves oral function and aesthetics. However, alternative reconstructive options may be more appropriate in cases where local tissue is insufficient or wound tension is high.

At the six-month follow-up, the color and texture match of the adjacent tissue resulted in an excellent cosmetic outcome, and with proper postoperative care, wound-healing complications were avoided. This case reinforces the importance of individualized, multilayered tension management in complex lip reconstruction.

## Conclusions

Complex surgical defects of the face, particularly the lips, can be challenging to reconstruct. When selecting a reconstruction approach, special consideration must be given to preserving both form and function. While MMS defects of the lips present unique challenges due to this area's functional and cosmetic significance, this case demonstrates that thoughtful planning and technique can achieve excellent outcomes. The use of an inverted subdermal-muscular plication layer combined with a mucocutaneous advancement, performed without a sulcal incision or backcut, provided a tension-free closure while maintaining lip integrity. This method avoided the need for secondary donor sites and minimized postoperative complications. Ultimately, a tailored approach that prioritizes both functional preservation and aesthetic outcome can offer patients a successful recovery, even in the setting of extensive defects.
